# Headache

**Published:** 1899-11-11

**Authors:** 


### HEADACHE.

Dr. Lauder Brunton[1] says that the headache which is associated with various nerve conditions and is known as migraine is probably almost entirely due to the stretching of the nerve fibres within the blood vessels. What, however, may be the immediate cause of this condition may be doubtful, but it seems clear that in its production two things, at any rate, have to be considered: (1) A general condition rendering the patient liable to pain, and (2) a local condition determining that the pain shall affect the head rather than any other part of the body. To take the general conditions first, they are those either of imperfect or of disordered nutrition. Headache is common enough in imperfect nutrition, such as anæmia, and perhaps still more common in disordered nutrition such as occurs in rheumatism, in gout, and above all in albuminuria. In all these cases the tendency to headache is more or less constant, because the disorder of nutrition is more or less permanent. But in apparently healthy people we find headaches come on now and again with much regularity, and it would appear that the regularly recurring disorder of nutrition by which they are produced is in some way connected with the liver. The liver, says Dr. Lauder Brunton, "is a porter which stands at the gate of the organism, and prevents all the deleterious substances which pass into the intestinal vessels from the intestine from reaching the general circulation." These substances are caught by the liver, where they are either transformed or destroyed, or are excreted back again into the intestine. When so poured back some of these substances may pass away with the fæcal matters, but many may be reabsorbed, "and so they go on in a continual round, from the intestine to the liver, from the liver to the intestine, and back again to the liver, until at last the amount of these substances becomes so great that the liver is no longer able to deal with it, and they pass through the liver, and get on into the general circulation." Here, then, we have the key to the recurrent headaches from which some people suffer. The period of recurrence will vary in different individuals, and even in the same individual under different circumstances, the tendency of the liver to fail as a filter being greatly increased by emotion, and by any excess of animal food. Why in such cases headache is produced instead of aches in other organs is probably determined by the presence of some disorder rendering the head a vulnerable part. Hence the necessity of investigating the eyes, the teeth, the nose and its accessory sinuses, the antrum and the upper pharynx. In discussing treatment one has to consider, on the one hand, these local causes by which the pain is attracted to the head rather than elsewhere; and, on the other, those conditions of liver and intestine on which depend the toxic condition of the blood. Clearly one means of relieving the latter is to remove from the intestines the morbid matters which form the source of the toxic materials, and this can be done, often with great effect, by the administration of a blue pill over night and a black draught the following morning. Then, again, by strictly limiting the amount of animal or nitrogenous food much good may be done, for it is probably by the decomposition of albuminous food in the intestines that most of these toxins are formed. But beyond such measures, we may try to give something which may have the power of counteracting these toxins or of eliminating them from the liver, and for this purpose we may administer salicylate of soda, "15, 20, or 30 grains at night, with 10, 20, or 30 grains of bromide of potassium. This mixture acts better than either salicylate of soda or bromide of potassium alone, and it will usually prevent the occurrence of a headache in the morning." If, however, the headache should still come on, the dose should be repeated, or the salicylate may be given in smaller doses three times a day. To prevent the depression which is apt to attend its use, this may be combined with half a drachm of spirit of ammonia, and if it should be necessary to continue the drug for long a little iron may be added to counteract its reputed tendency to produce anæmia. Then there are other drugs which tend to disperse pain, such as phenacetin, antipyrin, nux vomica, strychnine, and caffeine. All of these should be given before the pain becomes severe or they will not act, and may, indeed, aggravate the distress. Then we have such remedies as the hypodermic use of morphine, or the administration of cannabis indica, which is not dangerous even if run up to 15 or 20 minims, although an attack of maniacal delirium may in some cases be brought on. Finally, for the relief of headache which is due to inflammation of the periosteum, from gouty, rheumatic, or syphilitic irritation, there is iodide of potassium, which may be gradually increased up to 30 grains three times a day. As a last word, Dr. Brunton gives the warning to look out for glaucoma in all cases of severe headache which do not yield to treatment.

[1] Brit. Med. Jour., Nov. 4.

